# Feed Force and Sawdust Geometry in Particleboard Sawing

**DOI:** 10.3390/ma14040945

**Published:** 2021-02-17

**Authors:** Bartosz Pałubicki, Luďka Hlásková, Stephan Frömel-Frybort, Tomasz Rogoziński

**Affiliations:** 1 Department of Woodworking Machines and Fundamentals of Machine Design, Faculty of Forestry and Wood Technology, Poznań University of Life Sciences, ul. Wojska Polskiego 38-42, 60-637 Poznań, Poland; 2 Department of Wood Science and Technology, Faculty of Forestry and Wood Technology, Mendel University in Brno, Zemědělská 3, 61300 Brno, Czech Republic; ludka.hlaskova@mendelu.cz; 3 Wood K plus, Competence Centre for Wood Composites and Wood Chemistry, 4040 Linz, Austria; s.froemel-frybort@wood-kplus.at; 4 Department of Furniture Design, Faculty of Forestry and Wood Technology, Poznań University of Life Sciences, ul. Wojska Polskiego 38-42, 60-637 Poznań, Poland; tomasz.rogozinski@up.poznan.pl

**Keywords:** feed force, circular saw, particleboard, sawdust particle size distribution

## Abstract

The measurement of cutting forces permits building of physic-mechanical cutting models for a better understanding of the phenomena observed during cutting. It also permits the design and optimization of processes, machines, tools, and wood preparation. Optimization of cutting conditions of wood-based materials can decrease the cutting forces, which directly relates to the energy consumption and surface quality. The sawdust analysis may serve for analysis of cutting kinematics and occupational health risk. The aim of the study was to estimate the correlation between the feed rate and both feed force and sawdust particle size produced during particleboard circular sawing. A saw machine type K700 was used in experiments. There were three feed rates of 12, 18, and 24 m/min executed by a horizontal pneumatic actuator fixed to the sliding side table of the saw machine. Based on the results of the experiments, a positive correlation was observed between the feed rate in a circular sawing process and feed forces and an unexpected particle size distribution depending on the feed rate.

## 1. Introduction

At the time of rapid development of new materials, there is a growing need for a better understanding of the interaction between the workpiece and tool. Given that determination of the cutting forces is one of the basic criteria for machinability evaluation, accurate prediction of the cutting forces is a necessity. During the analysis of the cutting process, the cutting forces are very often chosen as the main outputs for the description of the cutting process. According Marchal et al. (2009) [1], the main reason of such an approach is that measurement of cutting force is a simple and useful tool to create the physico-mechanical cutting models that allow an explanation of the phenomena arising during machining. Using these models, it is possible to design or optimize machines, tools and cutting conditions. Moreover, optimizing wood cutting processes can reduce the value of cutting forces that are directly related to the energy consumption and surface quality.

According Thibaut et al. (2016) [2], the total force exerted by the tool on the workpiece can be decomposed using different geometrical sums into two components: the first is the cutting force and the thrust force, respectively, the parallel and the perpendicular forces to the cutting direction; and the second is the rake force and the clearance force, respectively, the resultant forces exerted by the tool rake face to remove chips and by the tool clearance face by friction against the workpiece just behind the tool tip.

The cutting forces exploration began in the 1950s and various empirical, statistical, and numerical methods have been proposed for their qualified estimation. The first systematic study of cutting forces during low-speed cutting of wood with different grain orientations and different chip types and formation was conducted in 1950 by Kivimaa [3], who developed a new method for dividing the cutting force into two components: the normal cutting force and the thrust cutting force [4]. More recent research examines the effects of moisture content [5] and wood density [5,6] on the cutting forces. Based on their research, Cristóvão et al. (2012) [5] provided the following conclusions—wood density and moisture content showed the lowest effect on the main cutting force for both species of tropical wood specimen. On the other hand, Axelsson et al. (1993) have drawn a conclusion that the cutting force increases with the density, and this statement was confirmed by other authors, e.g., Kivimaa (1950) [3] has reached the same conclusions.

Loehnertz and Cooz (1998) [7] recorded saw tooth cutting, thrust, and side forces for many hardwood species. Moradpour et al. (2016) [8] established that cutting forces are dependent on the cutting speed and feed rate. As the feed rate increased, the cutting forces also increased. Lucic et al. (2004) [9] showed the results of cutting power in relation to feed rate for three depths of cut for green, dried and frozen wood samples. Based on their results, it is obvious that the cutting force increases by increasing the feed rate and this growth could be expressed as linear. Heisel et al. (2007) specified that with increasing of feed rate, the normal and parallel force increases. Many other authors showed that the cutting force increases with feed rate (or feed force) [8,10,11,12,13]. Dippon et al. (2000) [14] discussed the cutting forces during machining MDF (Medium Density Fiberboard) where the cutting force and feed force were expressed as functions of cutting speed, feed rate, cutting depth and rake angle (tool geometry) [15]. Cutting forces were often measured to compare the cutting properties of different types of wood-based materials; for example, Kowaluk et al. (2004) [16] investigated the cutting forces during machining of modified MDF.

A review of the literature shows that the effect of cutting condition on the magnitudes of cutting forces, surface quality and chip formation are the main subjects of cutting studies. Forces acting on the workpiece and the tool in wood machining processes have been extensively investigated both theoretically and experimentally [1,17,18,19,20,21,22,23,24,25,26,27,28,29,30,31,32,33,34]. Nevertheless, none of these studies have examined the effect of the feed force, even though this is one of the most available parameters of the cutting process—easily sensed by the operator’s hand.

Moreover, the sawdust may serve as an accessible trace of the process. Theoretical chip shape and size when sawing can be derived with the use of analysis of cutting kinematics. In the case of inhomogeneous materials like particleboard, each of the theoretical chips consists of many smaller parts created from particles forming the board structure. This usually implies a noncoherent chip structure leading to its additional disintegration. This secondary wood fragmentation during particleboard sawing is a reason for the serious risk of dustiness. The smallest chips created during machining can be dispersed in the air surrounding a saw machine. Kos et al. (2004) [35] reported that Croatian limit values for respirable particles and total wood dust at woodworking places of circular saws when particleboard machining have been exceeded. A similar conclusion was made in the studies regarding the Japanese occupational exposure limit of respirable dust concentration [36].

Hlásková et al. (2016) [37] concluded that the rate of fine dust created when sawing double-sided laminated particleboards significantly increases with decreasing feed rate and therefore also feed per tooth. The phenomenon of finer dust creation during particleboard machining compared to other wood materials has been confirmed not only in the study reports on sawing. Milling, drilling, and sanding of particleboards are also connected with fine dust particle creation. In general, in the woodworking industry, dust particles finer than in the case of particleboard are formed only during the processing of fiberboard, especially MDF [38,39,40,41,42,43,44].

For this reason, attention should be paid to the cut chip geometry when machining particleboards. The sawdust geometry analysis can be therefore a way to determine the right machining parameters for the reduction of energy consumption (cutting force, feed force) on one hand and health risk on the other. The aim of the study was to determine the influence of the feed rate on the feed force, considered together with progressing tool wear and the sawdust geometry when particleboard sawing.

## 2. Materials and Methods

Commercially available, melamine-coated, 3-layered, *h* = 18 mm thickness particleboard panels produced by Kronospan (Szczecinek, Poland) company have been used as material for the experiments. The panels were produced from pine wood with the addition of laminated particle wastes with a urea-formaldehyde resin. Edges of the board sheets were trimmed to remove loose structure material. A total of 33 pieces of 500 × 500 mm^2^ panels have been prepared as specimens for the experiment. The average density of panels was 685 ± 21 kg/m^3^.

In the experiment, a saw blade of 300 mm in diameter (Pilana, Hulín, Czech Republic) with kerf thickness 3.2 mm, body thickness 2.2 mm equipped with 96 teeth grouped into trapezoidal-straight pairs was used. The rake angle of each tooth was 6°, sharpness angle 66° and clearance angle 18°. The saw was brand new at the beginning of the experiment and was resharpened by grinding after completing each constant feed rate cycle (150 m of kerf).

A 1.1 kW Felder K700 (Hall in Tirol, Austria) formatting table saw (circular) was used in the tests. A nominal spindle rotational speed was 4800 rpm, while the measured real value was *n* = 4720 rpm. The feed system consisted of a horizontal pneumatic actuator fixed to the sliding side table of the saw with the use of a piezoelectric force sensor (max load 1 kN). The double-action actuator had a 1200 mm stroke and cylinder bore diameter of 32 mm. It was powered by 0.6 MPa air pressure and the exhaust air dumping setup has been applied in order to maintain a stable feed rate. During the single test, the workpiece was firmly fixed to the saw’s sliding table by an eccentric clamp. It is assumed that the sliding table fixed to the machine’s body with ball bearing runners operated with negligible friction. Signal from the extensometer was amplified by an ADAM 3016 strain gauge (Advantech, Cincinnati, OH, USA) and digitalized by an analog-digital converter LabJack U6 (LabJack, Lakewood, CO, USA) with a sampling frequency of 20 kHz and 16-bit resolution. Results have been saved as voltage (V) values and converted to force (N) with the use of a previously acquired calibration function. 

**Figure 1:**
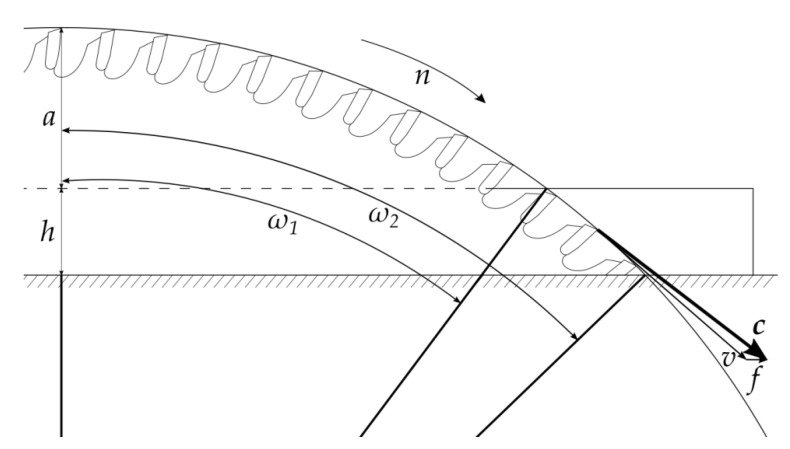
Sawing experiment setup: *a*—saw projection beyond the workpiece, *h*—thickness of particleboard, *ω*_1_—saw enter angle, *ω*_2_—saw exit angle, *n*—rotation of saw, *c*—peripheral velocity of saw (tooth), *f*—feed rate, *v*—real cutting velocity.

The workpiece sawing setup is presented in Figure 1. Three levels of feed rate have been utilized in examination: *f*_12_ = 12 m/min, *f*_18_ = 18 m/min, and *f*_24_ = 24 m/min. Peripheral velocity of the saw teeth for a constant saw radius (*R* = 150 mm) and rotational speed (*n*) was equal *c* = 74.1 m/s. The real cutting velocity (*v*), increased even by the highest feed rate (*f*_24_), was only slightly higher at *v*_24_ = 74.5 m/s. Since the saw had 48 teeth of each type, the feed per tooth (*ft*) for one tooth-type and for three feed rates was: *ft*_12_ = 0.053 mm, *ft*_18_ = 0.079 mm, *ft*_24_ = 0.106 mm. The saw projection beyond the workpiece was *a* = 30 mm.

**Figure 2:**
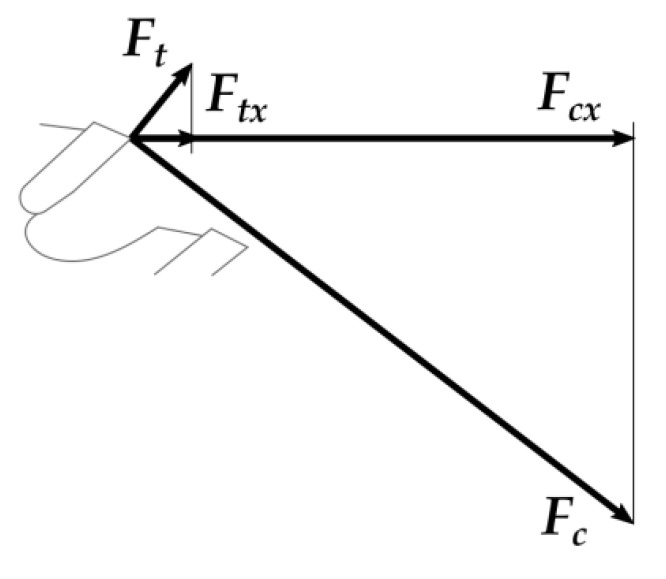
Forces in sawing: *F_c_*—cutting force, *F_t_*—cutting thrust force, and their horizontal projections: *F_cx_, F_tx_.*

During sawing with the current setup on the workpiece height (*h*) and with the saw projection beyond the workpiece *a* = 30 mm—2 or 3 teeth were involved in the cutting process at each moment. Alternating numbers and types of teeth involved in cutting, together with a variable theoretical chip thickness and heterogeneous structure of the panel, cause fluctuation of forces. Force vectors shown in Figure 2 symbolize the average forces reduced to one tooth placed in the middle of the workpiece thickness. Usually, when considering forces in cutting, for simplicity, it is assumed that Newton’s third law is applicable and there is a balance between all forces exerted on the tool and a workpiece [11,45]. This assumption is made also for the current analysis. Two forces acting on the workpiece are considered: a cutting force (*F_c_*) and a cutting thrust force (*F_t_*). Cutting thrust force may be positive or negative and is caused by chip interaction with the tooth, namely: pulling the workpiece onto the tool due to a stiff chip pressing on the rake face of the tooth, and the opposite force pushing it out due to compression between the workpiece and the clearance face of the tooth. In the case of current experiments: inhomogeneous particleboards, small chip thickness and small rake angle—it is positive, as shown in Figure 2.

The sum 1 of horizontal projections of the two mentioned forces (*F_cx_, F_tx_*) acts against the feed and its value equals the value of the resulting feed force (*F_f_*):

(1) $$F_{f}=F_{cx}+F_{tx}$$

The sawdust geometry analysis has been performed by means of the sieving analysis carried out using the Retsch AS 200 sieving machine (Retsch, Haan, Germany) according to the ISO 2591-1:1998 standard [46]. Six size classes and a corresponding set of five sieves with aperture sizes of: 1 mm, 500, 250, 125, 63 μm, and a collector of the remaining finer dust, was used. The arithmetic mean of particle sizes ($\bar{x}$) was calculated based on the results of the sieving analysis with use of Formula (2):

(2) $$\bar{x}=\;\sum_{i=1}^{6}x_{i}\cdot \;q_{3}{}_{i},$$

where:

*x_i_*—mean value of particle size in *i*-class [μm],
*q*_3*i*_—particle distribution by mass [%].

The obtained results of size classes (particle distributions) as well as the arithmetic means of particle sizes have been correlated to the feed rates used and the mean feed forces with use the analysis of variance (ANOVA).

## 3. Results and Discussion

**Figure 3:**
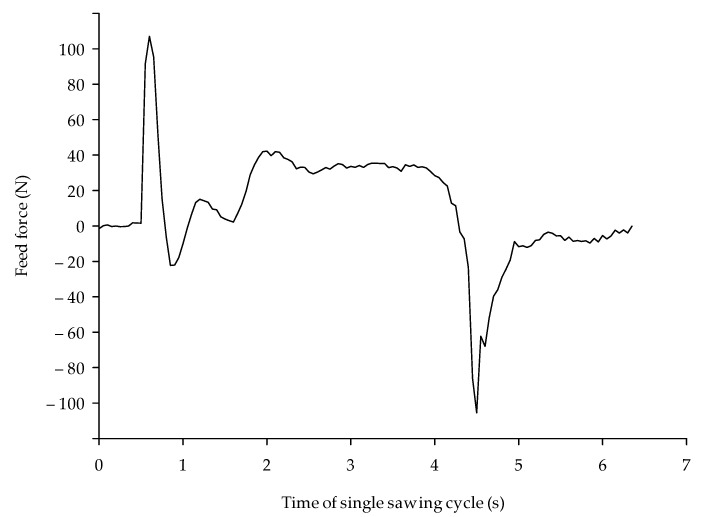
Force signal for a single sawing cycle for the 12 m/s feed rate.

In Figure 3, a single test cycle force signal is presented. The first positive force peak is caused by an acceleration of the saw’s sliding table with the workpiece from zero to the feed rate (12 m/s). Then, a single fluctuation with stabilization (dumping) is visible. Next, the increase of the force starting around 1.6 s shows the beginning of the sawing process. Then, after a small peak, the signal stabilizes, as there are always at least two teeth in contact with the workpiece, leading to a continuous signal. For the calculation of the mean feed force value, only this so called steady-state signal range from each single measurement was taken into consideration, in this case between 2.8 and 3.8 s. The following negative peak (−100 N) is a consequence of the deceleration of the table carrying the workpiece, caused by the reversion of the actuator movement.

**Figure 4:**
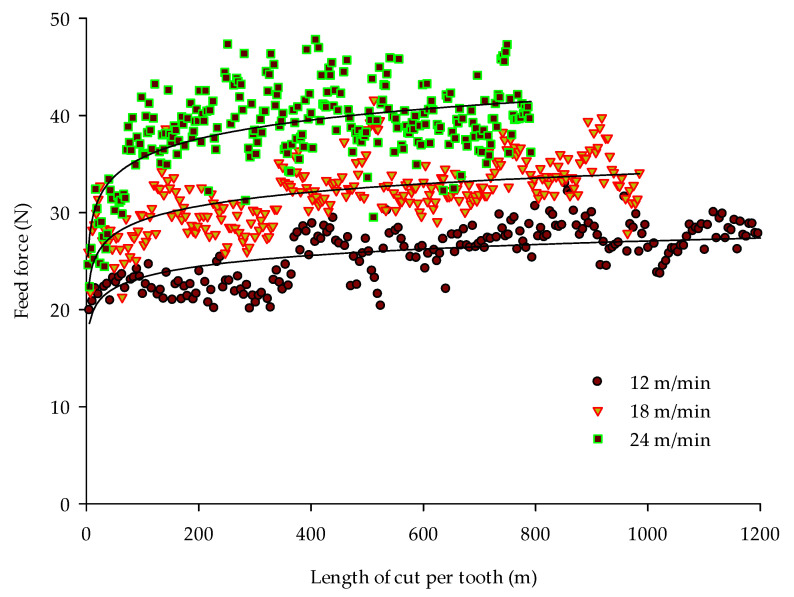
Feed forces for different lengths of cut per tooth.

The steady-state mean feed forces (*F_f_*) for three feed rates changing with the progressing real length of cut (*L_t_*) made by each tooth of the saw are shown in Figure 4. Each of the three cases were tested with the same length of kerf (150 m), but the total length of cut was different because of different feed per tooth values. The logarithmic regression equations of feed forces (given in N) for 12, 18, and 24 m/min feed rates, as functions of the length of cut (given in meters), are as follows:

(3) $$F_{f12}=1.62\ln\left(L_{t}\right)+15.87\;\;\;\;\;\;\;\;\;\;\;\;\;\;\;r^{2}=0.43$$

(4) $$F_{f18}=2.09\ln\left(L_{t}\right)+19.59\;\;\;\;\;\;\;\;\;\;\;\;\;\;\;r^{2}=0.38$$

(5) $$F_{f24}=2.79\ln\left(L_{t}\right)+22.82\;\;\;\;\;\;\;\;\;\;\;\;\;\;\;r^{2}=0.34$$

It is obvious that the force required to move the workpiece against the saw blade with the speed 12–24 m/min ranges for current experiments from around 20 up to 50 N. In general, the force increases for all feed rate cases with the length of cut, what is an effect of progressing wear of the saw’s teeth. Interestingly, it can be observed that with a perfectly sharp knife, the feed forces differ between feed rates only by 3–4 N. After short cutting, the forces at each feed rate achieve twice as much difference (6–8 N). The faster initial increase of feed force with increasing feed rate is most likely related to an accelerated tool wear which is connected to an increased feed per tooth. While the lowest feed rate leads to a relatively slow and linear increase of the feed forces, at the highest feed rate, an excessive increase of the feed force is initially observed.

Because of the tool dulling during the sawing of 150 m (kerf) of the particleboards, the feed force that was needed to push the workpiece against the saw increased by a similar value of around 75% of the initial force for the sharp saw. The higher the feed rate the higher the force increase; for *f*_12_ the feed force increased by 72%, for *f*_18_—74%, while for *f*_24_ the augmentation of the feed force due to tool blunting achieved 81%.

The coefficients of determination of Equations (3–5) are quite low and decreases from 0.43 to 0.34 with increasing feed rate. This is because of a higher dynamic of the workpiece movement against the saw, which leads to a higher initial force peak when the contact starts. Additionally, the sawing cycle is shorter for higher feed rates and therefore less time is available for achieving the steady-state force signal and as a consequence, higher fluctuations are observed.

**Figure 5:**
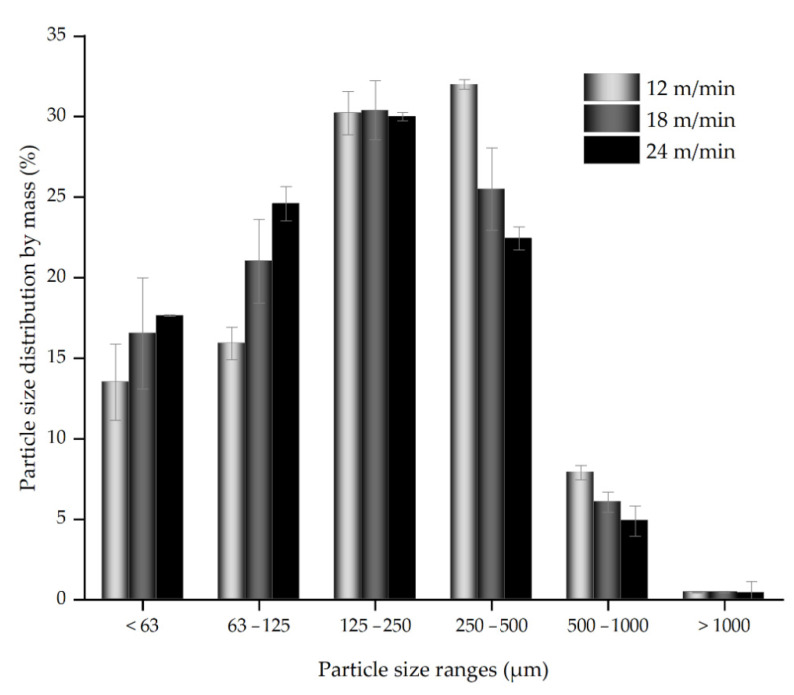
Particle size distribution by mass.

In Figure 5, the particle size distribution of dust is presented. The dust particles of mid-size classes (125–250 and 250–500 μm) have the highest shares. Comparing the mass shares of six size classes for different feed rates, one will notice an interesting relation. The amount of particles smaller than 125 μm increases when increasing the sawing feed rate, and on the contrary, faster feeding produces smaller particles (250–500 and 500–1000 μm).

This is quite unexpected, since, as concluded by Hlásková et al. (2016) [37], the smaller feed rate denotes smaller feed per tooth and therefore smaller theoretical chip thickness (assuming the other dimensions are constant). The theoretical chip cut of particleboard consists of numerous smaller particles sliced from chips creating the particleboard’s structure. Nevertheless, these sliced particles were expected to be generally bigger for 24 m/min of feed rate than for 12 m/min. A possible explanation of this phenomenon lies in a mechanism of cutting with a blunt tool. In the mentioned report [37], the tool used was sharp (mean tooth tip rounding r = 9 μm) during the whole experiment, since only several meters of kerf were sawn. In the present experiment, in order to find the cause of the results discrepancy, the wear of teeth were measured after the cutting experiment with use of a Stemi DV4 (Carl Zeiss, Jena, Germany) microscope. The final rounding of teeth reached a level of r = 60 μm. For feed per tooth of *ft*_12_ = 53 μm, smaller than the rounding of teeth cutting edges (later phase of sawing experiment), the cutting becomes more rubbing than splitting. Rubbing, in the case of noncontinuous material like a particleboard, may lead to ripping out bigger chips from the panel structure instead of cutting smaller slices out of them. This effect becomes less relevant with increasing feed per tooth values and leads to a lower share of big particles. As the tool wear plays an important role in stress concentration during the cutting process and leads to different disintegration of the sawn material, results of these two experiments are not comparable.

Another possible explanation is that an increasing chip size leads to an increasing tendency of an additional disintegration of the freshly cut chips. This effect could be related to the increased stiffness of the thicker chips, which have comparably less space to be removed out of the saw kerf and are less likely to be bent during their movement out of the saw kerf.

The calculated arithmetic mean values of particle sizes for the three feed rates are as follows: $\bar{x}$_12_ = 262 μm, $\bar{x}$_18_ = 230 μm, $\bar{x}$_24_ = 212 μm. These values decrease with increasing feed rate, what confirms the conclusions from the previous discussion.

Correlations between the six particle size ranges, the arithmetic means of particle sizes on the one hand, and feed rate and mean feed force on the other, are shown in Table 1. The mean feed force shows the highest positive correlation with the smallest particle shear, meaning that when higher feed forces were required to cut the material, one may expect more fine dust to be generated during the process. On the contrary, the amount (share) of 250–500 μm particles has a high inverse correlation to the mean feed force, therefore less particles of this size may be expected when higher feed force had to be exerted during particleboard sawing. For the feed rate, a better correlation exists to 63–125 μm particle size share (positive) and to the amount of 500–1000 μm chips. Only particles ranging from 125 to 250 µm, as well as above 1000 µm, show no significant correlation with feed rate and feed force. Almost identical and good correlation (negative) was found for the arithmetic mean of particle sizes to both feed rate and feed force.

## 4. Conclusions

Within this study, the positive correlation between the feed rate in a circular sawing process and feed forces could be observed. Additionally, increased feed forces due to blunting of the tools could be noticed, whereby a faster dulling was observed at higher feed rates. However, no significant differences between the initial feed forces at the beginning of each examination, i.e., using a freshly sharpened saw blade, were observed with respect to different feed rates. The study showed that it is possible to bypass the complex method of direct cutting force measurement by deriving the feed force. This also allowed relatively subtle differences resulting from the initial wear and the uniform wear rate to be observed.

An unexpected particle size distribution depending on the feed rate was observed. In contrast to the results known from the literature, an increasing fine fraction share was observed with increasing feed rate. This unexpected negative correlation between feed rate and particle size is probably caused by high tool wear compared to feed per tooth, and it requires further investigation, as particle size, especially particulate matter, is a crucial factor regarding workplace safety. With respect to tool wear, as a result of cutting forces, the lowest or medium feed rate of 12 and 18 m/min, respectively, is to be preferred. With respect to the particle size distribution, the lowest feed rate of 12 m/min is to be preferred, resulting in a lesser quantity of hazardous fine dust.

## Figures and Tables

**Table 1:** Pearson’s correlation coefficients (** denote the highest correlation, *—other statistically significant at the level of significance α = 0.05).

	Particle Size Range [μm]						Arithmetic Mean of Particle Sizes	Mean Feed Force
	<63	63–125	125–250	250–500	500–1000	≥1000		
**Feed rate**	0.965 *	0.995 **	−0.561	−0.979 *	−0.992 *	−0.717	−0.987 *	0.949
**Mean feed force**	0.998 **	0.976 *	−0.270	−0.993 *	−0.981 *	−0.460	−0.987 *	x

## Data Availability

Data available at: https://doi.org/10.6084/m9.figshare.14043698.v1.
